# The Brm-HDAC3-Erm repressor complex suppresses dedifferentiation in *Drosophila* type II neuroblast lineages

**DOI:** 10.7554/eLife.01906

**Published:** 2014-03-11

**Authors:** Chwee Tat Koe, Song Li, Fabrizio Rossi, Jack Jing Lin Wong, Yan Wang, Zhizhuo Zhang, Keng Chen, Sherry Shiying Aw, Helena E Richardson, Paul Robson, Wing-Kin Sung, Fengwei Yu, Cayetano Gonzalez, Hongyan Wang

**Affiliations:** 1 Neuroscience and Behavioral Disorders Program, Duke-NUS Graduate Medical School Singapore, Singapore, Singapore; 2 NUS Graduate School for Integrative Sciences and Engineering, National University of Singapore, Singapore, Singapore; 3 Cell Division Group, Institute for Research in Biomedicine, Barcelona, Spain; 4 Temasek Life Sciences Laboratory, Singapore, Singapore; 5 Department of Computer Science, National University of Singapore, Singapore, Singapore; 6 Peter MacCallum Cancer Centre, East Melbourne, Australia; 7 Biochemistry and Molecular Biology Department, University of Melbourne, Parkville, Australia; 8 Genome Institute of Singapore, Singapore, Singapore; 9 Department of Biological Sciences, National University of Singapore, Singapore, Singapore; 10 Institució Catalana de Recerca i Estudis Avançats (ICREA), Barcelona, Spain; 11 Department of Physiology, Yong Loo Lin School of Medicine, National University of Singapore, Singapore, Singapore; 12 Sir Peter MacCallum Department of Oncology, University of Melbourne, Parkville, Australia; 13 Anatomy and Neuroscience Department, University of Melbourne, Parkville, Australia; University of Cambridge, United Kingdom

**Keywords:** neuroblast, self-renewal, differentiation, dedifferentiation, intermediate neural progenitor, Drosophila, *D. melanogaster*

## Abstract

The control of self-renewal and differentiation of neural stem and progenitor cells is a crucial issue in stem cell and cancer biology. *Drosophila* type II neuroblast lineages are prone to developing impaired neuroblast homeostasis if the limited self-renewing potential of intermediate neural progenitors (INPs) is unrestrained. Here, we demonstrate that *Drosophila* SWI/SNF chromatin remodeling Brahma (Brm) complex functions cooperatively with another chromatin remodeling factor, Histone deacetylase 3 (HDAC3) to suppress the formation of ectopic type II neuroblasts. We show that multiple components of the Brm complex and HDAC3 physically associate with Earmuff (Erm), a type II-specific transcription factor that prevents dedifferentiation of INPs into neuroblasts. Consistently, the predicted Erm-binding motif is present in most of known binding loci of Brm. Furthermore, *brm* and *hdac3* genetically interact with *erm* to prevent type II neuroblast overgrowth. Thus, the Brm-HDAC3-Erm repressor complex suppresses dedifferentiation of INPs back into type II neuroblasts.

**DOI:** http://dx.doi.org/10.7554/eLife.01906.001

## eLife digest

Stem cells show great promise for repairing damaged tissue, and maybe even generating new organs, but stem cell therapies will only be successful if researchers can understand and control the behaviour of stem cells in the lab. Neural stem cells or ‘neuroblasts’ from the brains of larval fruit flies have become a popular model for studying these processes, and one type of neuroblast—known as a ‘type II’ neuroblast—is similar to mammalian neural stem cells in many ways.

When type II neuroblasts divide, they generate another neuroblast and a second cell called an intermediate neural progenitor (INP) cell. This progenitor cell then matures and undergoes a limited number of divisions to generate more INP cells and cells called ganglion mother cells. The process by which stem cells and INP cells become specific types of cells is known as differentiation. However, under certain circumstances, the INP cells can undergo the opposite process, which is called dedifferentiation, and become ‘ectopic neuroblasts’. This can give rise to tumors, so cells must employ a mechanism to prevent dedifferentiation. Researchers have known that a protein specifically expressed in INP cells called Earmuff is involved in this process, but many of the details have remained hidden.

Now, Koe et al. have discovered that a multi-protein complex containing Earmuff and a number of other proteins—Brahma and HDAC3—have important roles in preventing dedifferentiation. All three proteins are involved in different aspects of gene expression: Earmuff is a transcription factor that controls the process by which the genes in DNA are transcribed to make molecules of messenger RNA; Brahma and HDAC3 are both involved in a process called chromatin remodeling. The DNA inside cells is packaged into a compact structure known as chromatin, and chromatin remodeling involves partially unpacking this structure so that transcription factors and other proteins can have access to the DNA.

Koe et al. also showed that Earmuff, Brahma and HDAC3 combine to form a complex that prevents dedifferentiation. An immediate priority is to identify those genes whose expression is regulated by this complex in order to prevent dedifferentiation.

**DOI:** http://dx.doi.org/10.7554/eLife.01906.002

## Introduction

The mechanism by which self-renewal and differentiation are balanced is a crucial issue in stem cell and cancer biology. The neural stem cells, or neuroblasts, of the *Drosophila* larval brain have emerged as a new model for studying stem cell self-renewal and tumorigenesis. In *Drosophila* larval central brains, there are at least two classes of neuroblast lineages (Bello et al., 2008; Boone and Doe, 2008; Bowman et al., 2008). A type I neuroblast that expresses both Deadpan (Dpn) and Asense (Ase) divides asymmetrically to generate a self-renewing neuroblast and a ganglion mother cell (GMC), which is committed to a differentiation pathway. In contrast, a type II neuroblast that expresses Dpn, but not Ase, divides asymmetrically to generate a neuroblast and a transient amplifying cell known as an intermediate neural progenitor (INP) (Bello et al., 2008; Boone and Doe, 2008; Bowman et al., 2008). Following maturation, the INP undergoes a limited number of asymmetric divisions to self-renew and to produce multiple GMCs (Weng et al., 2010). In both types of lineages, asymmetric division is dependent on apically localized proteins, including atypical protein kinase C (aPKC); basally localized proteins, such as Miranda and Numb; as well as several cell cycle regulators (Chang et al., 2012; Gonzalez, 2013). The failure of asymmetric division in either type of neuroblast can result in the hyperproliferation of these cells and the induction of brain tumors (Caussinus and Gonzalez, 2005; Wang et al., 2006, 2007, 2009, 2011; Lee et al., 2006a, 2006b; Cabernard and Doe, 2009; Chabu and Doe, 2009, 2011; Chang et al., 2010).

The type II neuroblast lineage is highly analogous to the mammalian neural stem cell lineages, because both involve transient amplifying cells that are used to expand the progenitor cell population. It is prone to impaired neuroblast homeostasis, if the limited self-renewing potential of INPs is unrestrained. Brain tumor (Brat) and the Notch antagonist Numb function cooperatively to ensure that immature INPs undergo maturation and commit to the INP fate (Boone and Doe, 2008; Bowman et al., 2008). Notch signaling maintains neuroblast identity and its overactivation leads to dedifferentiation of INPs to ectopic neuroblasts (Wang et al., 2006; Bowman et al., 2008; Weng et al., 2010). A small number of transcription factors have been implicated in the control of INP identity and proliferative potential (Carney et al., 2012). Specifically expressed in INPs, a Zinc-finger transcription factor Earmuff (Erm) plays a critical role in maintaining the restricted developmental potential of the INPs (Weng et al., 2010). The Ets transcription factor Pointed (PntP1) is specifically expressed in type II neuroblasts and INPs and is both necessary and sufficient for the suppression of Ase in type II neuroblasts and the generation of INPs (Zhu et al., 2011). Prospero that is basally localized in mitotic type I neuroblast, but absent from type II neuroblasts, triggers cell cycle exit and GMC differentiation (Bello et al., 2006; Betschinger et al., 2006; Choksi et al., 2006; Lee et al., 2006c). However, the underlying mechanism by which Erm prevents dedifferentiation is poorly understood.

ATP-dependent chromatin-remodeling factors are critical for the expression of the eukaryotic genome. Four major classes of ATP-dependent chromatin remodeling complexes have been identified, including the extensively studied SWI/SNF complexes (Narlikar et al., 2002; Reisman et al., 2009). The mammalian SWI/SNF complex termed the Brahma (Brm or Brg1) complex regulates critical cellular processes such as differentiation and cell cycle arrest (Klochendler-Yeivin et al., 2002). *Drosophila* Brm complex acts similarly to control cell proliferation (Brumby et al., 2002) and differentiation (Marenda et al., 2003). A genome-wide RNAi study in *Drosophila* neuroblasts showed that the knockdown of genes encoding several core subunits of the SWI/SNF Brahma (Brm) remodeling complex may lead to neuroblast overproliferation (Neumuller et al., 2011). However, the precise role of the Brm remodeling complex during neuroblast self-renewal and the mechanism that underlying underlies this effect mechanism remain to be elucidated. Besides ATP-dependent chromatin remodeling complexes, the other major class of chromatin remodelers is histone modifiers. Histone deacetylases (HDACs) remove acetyl groups from the tails of core histones in the nucleosome and are often associated with transcriptional co-repressors (Dokmanovic et al., 2007). However, despite the critical role for histone modifiers in transcriptional regulation, it is unknown whether histone modifications play any role in *Drosophila* larval brain neuroblasts.

In this study, we report the critical role of a central chromatin remodeler, the Brm complex in preventing the formation of ectopic neuroblasts in type II lineages. We show that another chromatin remodeling factor, HDAC3 functions cooperatively with the Brm complex to suppress the formation of ectopic type II neuroblasts. Interestingly, multiple components of the Brm complex and HDAC3 physically associate with Erm. *brm* and *hdac3* interact genetically with *erm* to prevent type II neuroblast overgrowth. Thus, the Brm-HDAC3-Erm complex is a novel repressor complex that suppresses dedifferentiation of INPs back into type II neuroblasts.

## Results

### The Brm complex suppresses the formation of ectopic neuroblasts in type II neuroblast lineages

**Figure 1.**
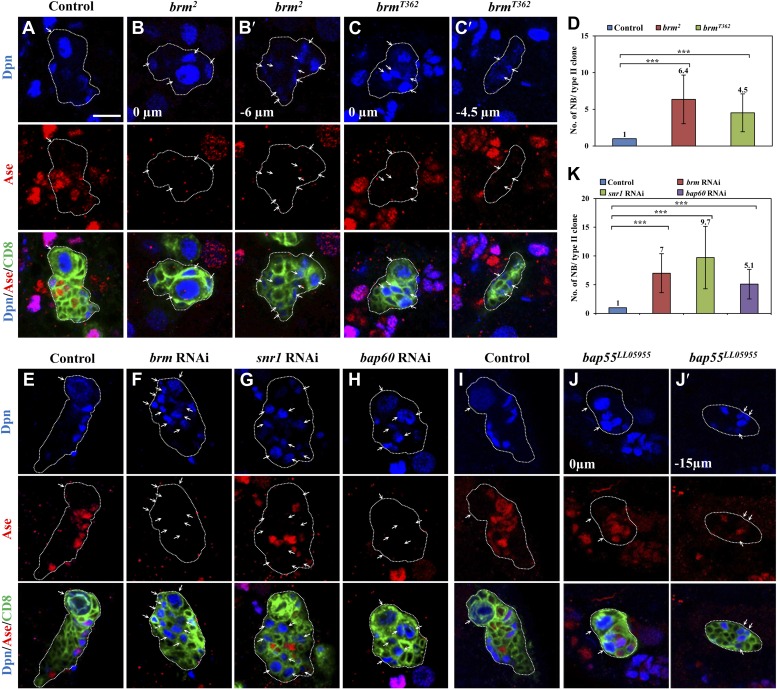
The Brm complex suppresses the formation of ectopic type II neuroblasts. (**A**–**C**) Type II MARCM clones of control (the MARCM driver; **D**), *brm*^*2*^ (**B**, **B′**) and *brm*^*T362*^ (**C**, **C′**) were labeled with Dpn (blue), Ase (red) and CD8::GFP (green). (**D**) Quantification of neuroblast number per type II MARCM clone for **A**–**C**. (**E**–**H**) Type II neuroblast lineage from control (‘the type II driver’: *wor*-Gal4 *ase-Gal80*; **E**), *brm* knockdown (**F**), *snr1* knockdown (108599 KK; **G**), and *bap60* knockdown (**H**) were labeled with Dpn (blue), Ase (red) and CD8 (green). (**I**–**J′**) type II MARCM clone of control (**I**) and *bap55*^*LL05955*^ (**J**, **J′**) were labeled with Dpn (blue), Ase (red) and CD8 (green). (**K**) Quantification of neuroblast number per type II lineage for **E**–**I**. Arrows indicate neuroblasts. Clones are marked by CD8::GFP and indicated by white dotted line. Scale bars, 10 µm. *** indicates p<0.001. **DOI:** http://dx.doi.org/10.7554/eLife.01906.003

We independently identified *brm* from a RNA interference (RNAi) screen in which *brm* RNAi knockdown in larval brains resulted in an increase of Miranda-positive neuroblast-like cells in larval central brains (Figure 1—figure supplement 1A), showing a phenotype similar to one previously reported (Neumuller et al., 2011). Brm is a DNA-dependent ATPase and a major component of a multi-protein SWI/SNF chromatin remodeling complex, which controls gene expression by altering chromatin structure (Klochendler-Yeivin et al., 2002). The number of cells expressing the proto-oncogene dMyc was significantly increased upon *brm* RNAi knockdown (Figure 1—figure supplement 1B), consistent with the neuroblast overgrowth phenotype. To determine the function of Brm in different neuroblast lineages, we generated MARCM clones in two *brm* loss-of-function alleles. Type I wild-type (wt) clones always contained one neuroblast that is positive for both Dpn and Ase (data not shown). Similarly, only one neuroblast was present in both amorphic *brm*^*2*^ and hypomorphic *brm*^*T362*^ type I clones (data not shown), indicating that Brm has no significant effect on type I neuroblast numbers. Each wt type II MARCM clone also possessed only one neuroblast that was positive for Dpn, but negative for Ase (Figure 1A; n = 25). Unlike the wt control, 6.4 ± 3.3 and 4.5 ± 2.6 ectopic neuroblasts were observed in *brm*^*2*^ (Figure 1B,B′,D; 88.6%, n = 34) *brm*^*T362*^ (Figure 1C,C′,D; 75.9%, n = 58) type II clones, respectively. These phenotypes in *brm* alleles could be rescued by expressing a wild-type *brm* transgene (Figure 1—figure supplement 1C). Consistent with phenotypes in *brm* clones, knockdown of *brm* by RNAi using a type II neuroblast-specific neuroblast driver *worniu* (*wor*)*-*Gal4, *ase*-Gal80 (henceforth referred to as ‘type II driver’; ‘Materials and methods’) was sufficient to produce ectopic neuroblasts in 97.4% of type II neuroblast lineages (Figure 1F,K; 7 ± 3.4 neuroblasts/lineage, n = 38), while each control clone always has one neuroblast (Figure 1E,K; n = 80). We therefore conclude that Brm suppresses the formation of ectopic neuroblasts in type II neuroblast lineages.

**Figure 1—figure supplement 1.**
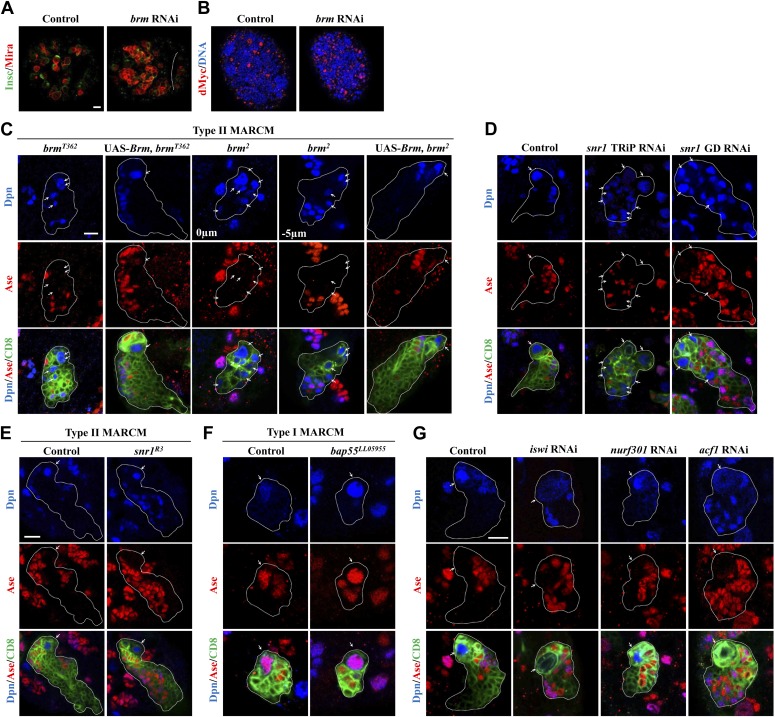
Analysis of chromatin remodelers in larval brains. (**A** and **B**) Larval brains of control (*elav*-Gal4 driver) and *brm* knockdown under the control of *elav*-Gal4 driver were labeled with Insc and Mira (**A**) and dMyc (**B**). Central brain is to the left of white dotted line. (**C**) Type II MARCM clones of *brm*^*T362*^, *brm*^*2*^ with or without the expression of UAS-Brm were labeled with Dpn, Ase and CD8. (**D**) Type II driver control, *snr1* TRiP RNAi (BDRC#32372), and *snr1* VDRC RNAi (12645GD) under the type II driver were labeled with Dpn, Ase and CD8. (**E**) Control (MARCM driver) and *snr1*^*R3*^ type II MARCM clones were labeled with Dpn, Ase and CD8. (**F**) Control (MARCM driver) and *bap55*^*LL5955*^ type I MARCM clones were labeled with Dpn, Ase and CD8. (**G**) Type II neuroblast lineages of control (type II driver), *iswi* knockdown, *nurf301* knockdown and *acf1* knockdown were labeled with Dpn, Ase and CD8. Arrows, neuroblasts. Clone outline is indicated by white dotted line (**C**–**G**). Scale bars, 10 μm. **DOI:** http://dx.doi.org/10.7554/eLife.01906.004

**Figure 2.**
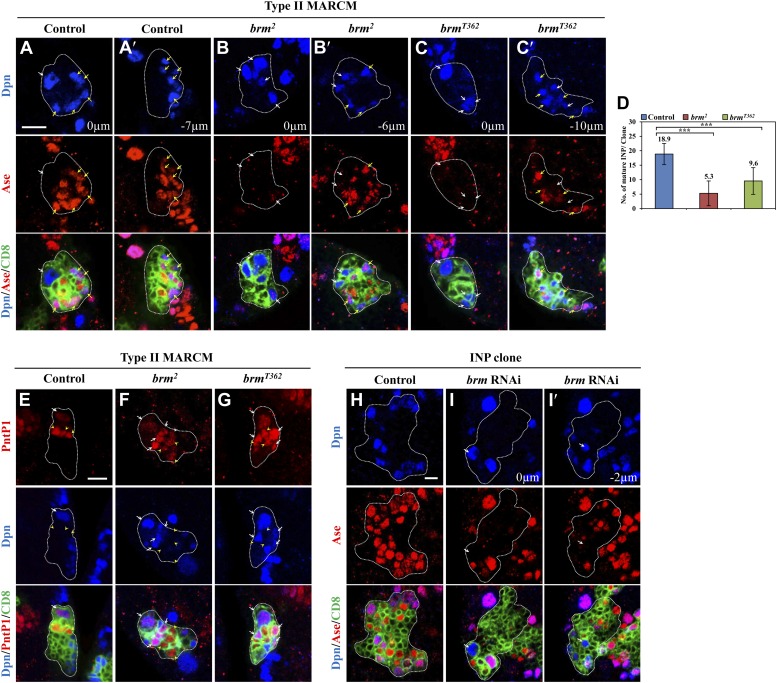
The Brm complex suppresses INP dedifferentiation into type II neuroblasts. (**A**–**C′**) Type II MARCM clones of control (the MARCM driver; **A**, **A′**), *brm*^*2*^ (**B**, **B′**) and *brm*^*T362*^ (**C**, **C′**) were labeled with Dpn (blue), Ase (red) and CD8::GFP (green). (**D**) Quantifications of INP number per type II clone for **A**–**C′**. *** indicates p<0.001. (**E**–**G**) Type II MARCM clones of control (**E**), *brm*^*2*^ (**F**) and *brm*^*T362*^ (**G**) were labeled with Dpn (blue), PntP1 (red) and CD8::GFP (green). (**H**–**I′**) INP clones of a control (driver: *erm*-Gal4 [II]; *erm*-Gal4 [III]; (**H**) and *brm* RNAi under *erm*-Gal4 (II); *erm*-Gal4 (III) with UAS-Dcr2 UAS-CD8-GFP (**I**, **I′**) were labeled with Dpn (blue), Ase (red) and CD8 (green). White arrows indicate neuroblasts, yellow arrows indicate Dpn^+^ Ase^+^ mature INPs and yellow arrowheads indicate Dpn^−^ PntP1^+^ INPs. Clones are marked by CD8::GFP and indicated by white dotted line. Scale bars, 10 µm (**A**–**G**) and 5 µm (**H**–**I′**). **DOI:** http://dx.doi.org/10.7554/eLife.01906.005

Concomitantly with the formation of supernumerary neuroblasts, the number of mature INPs that are positive for Dpn and Ase were dramatically reduced in both *brm*^*2*^ (Figure 2B,B′,D; 5.3 ± 4.3/clone, n = 39) and *brm*^*T362*^ (Figure 2C,C′,D; 9.6 ± 4.6/clone, n = 56), compared with the control type II clones (Figure 2A,A′,D; 18.9 ± 3.7, n = 22). The number of Dpn^−^ PntP1^+^ immature INPs and early mature INPs appeared to be normal or slightly increased in *brm*^*2*^ (Figure 2F; 8.4 ± 2.5, n = 26) and *brm*^*T362*^ (Figure 2G; 7.1 ± 2.1, n = 33) clones compared with the control (Figure 2E; 5.9 ± 1.1, n = 26). These results suggested that ectopic neuroblasts in *brm*^*−*^ clones likely originate from INPs that fail to undergo maturation. To further determine whether INPs undergo dedifferentiation back into neuroblast, we used INP-specific RNAi to knock down *brm* in INPs by *erm*-Gal4, an INP-specific driver. In 42.5% of INP clones with *brm* knockdown, ectopic Dpn^+^ Ase^−^ neuroblasts were observed (Figure 2I,I′; 1.2 ± 1.6 neuroblasts /INP clone, n = 40). In contrast, none of the INP clones from the driver control contained any neuroblasts (Figure 2H; 0 neuroblast/INP clone, n = 53). The relatively weak phenotype is likely due to incomplete knockdown of Brm, as shown by the reduced Brm staining in the INP clones (Figure 2—figure supplement 1). Thus, our data suggest that Brm functions in INPs to prevent INP dedifferentiation back into neuroblasts.

**Figure 2—figure supplement 1.**
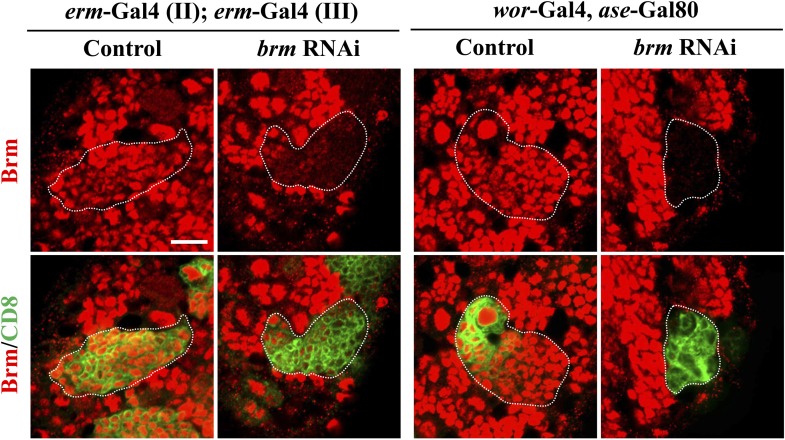
Partial knock down of *brm* in INP clones. Left panels, INP clones of a control (driver: *erm*-Gal4 [II]; *erm*-Gal4 [III]) and *brm* RNAi under *erm*-Gal4 (II); *erm*-Gal4 (III) with UAS-Dcr2 UAS-CD8-GFP were labeled with Brm (red) and CD8 (green). Right panels, Brm is absent in type II neuroblast clones under the type II driver. Clones are indicated by white dotted line. Scale bars, 10 µm. **DOI:** http://dx.doi.org/10.7554/eLife.01906.006

To assess whether Brm was affecting apico-basal cell polarity, we examined the localization of aPKC, Numb and Brat, which are asymmetrically localized in wild-type neuroblasts in prometaphase/metaphase. *brm*^*2*^ MARCM clones did not show any defects in the localization of these markers (Figure 3—figure supplement 1A–C), suggesting that Brm is not important for the apical-basal polarity regulation in neuroblasts.

We then assessed the function of other core subunits of the Brm remodeling complex using RNAi knockdown in larval brains. Knock down either of the three core components *snr1* (Figure 1G,K, 9.5 ± 5.5 neuroblasts/lineage, 64.5%, n = 34), *bap60* (Figure 1H,K, 5.1 ± 2.8 neuroblasts/lineage, 87.0%, n = 52), and *moira* (data not shown; also reported in Neumuller et al. (2011)) resulted in prominent phenotypes with ectopic Dpn^+^ Ase^−^ neuroblasts in type II neuroblast lineages. Two additional *snr1* RNAi lines under the control of the type II driver also displayed excess type II neuroblasts (Figure 1—figure supplement 1D). Furthermore, *bap55*^*LL5955*^ MARCM clones also showed ectopic neuroblasts in type II (Figure 1J,J′, 2.3 ± 1.7 neuroblasts/clone; 30.5%, n = 57), but not type I neuroblast lineages (Figure 1—figure supplement 1F; n = 20). The number of Dpn^+^ Ase^+^ mature INPs in *bap55*^*LL5955*^ was significantly reduced (13.8 ± 4.1; n = 49) compared with the control (18.9 ± 3.7, n = 22), while the number of Dpn^−^ PntP1^+^ immature and early mature INPs (4.2 ± 1.9, n = 26) was similar to the control (5.9 ± 1.1, n = 26). In various mutants and RNAi lines described above, we also observed an increased number of Dpn^+^ PntP1^+^ cells (data not shown), which serves as an independent set of marker for type II neuroblasts. This data further supports that loss-of-function of the Brm complex caused the phenotype of ectopic type II neuroblasts. We conclude that core components of the Brm remodeling complex are required to suppress ectopic neuroblast formation in type II neuroblast lineages.

Next, we ascertained whether other chromatin remodeling complexes such as Nucleosome remodeling factor (NURF) and ACF complex (ATP-utilizing chromatin assembly and remodeling factor), play any role during neuroblast self-renewal. RNAi knockdown of *nurf301* or ACF complex components *iswi* and *acf1* (Figure 1—figure supplement 1G) in the type II neuroblast lineages did not result in any obvious neuroblast overgrowth. These data suggest that they may not be important for type II neuroblast lineages or their RNAi targeting was insufficient to induce an effect.

### HDAC3 acts cooperatively with the Brm complex to suppress the formation of ectopic type II neuroblasts

**Figure 3.**
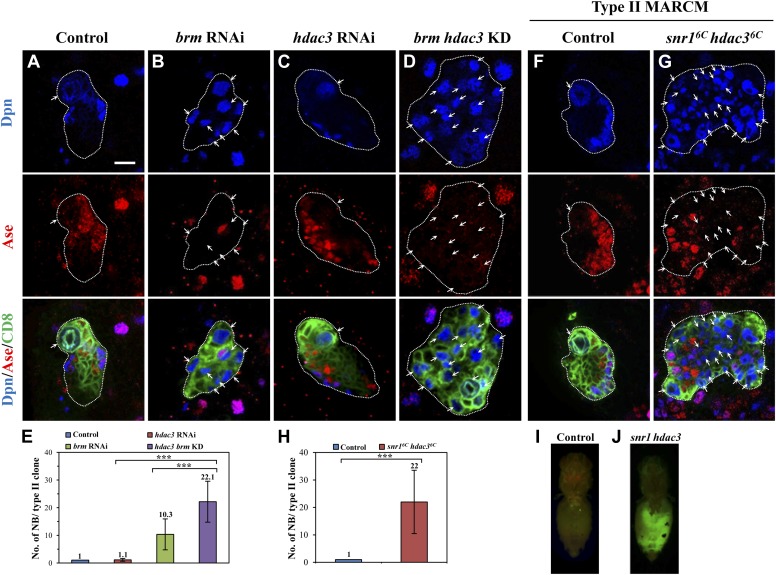
HDAC3 acts cooperatively with the Brm complex to suppress the formation of ectopic type II neuroblasts. (**A**–**D**) The driver control (**A**), *brm* RNAi (**B**), *hdac3* RNAi (**C**), *brm hdac3* double knockdown (**D**) under the type II driver were labeled with Dpn, Ase, and CD8. (**E**) Quantification of neuroblast number per type II MARCM clone in **A**–**D**. (**F**–**G**) Type II MARCM clones from the driver control (**F**) and *snr1*^*6c*^ *hdac3*^*6c*^ (**G**) homozygous MARCM clones were labeled with Dpn, Ase and CD8. Arrows indicate neuroblasts. (**H**) Quantification of neuroblast number per type II MARCM clone in **F**–**G**. *** indicates p<0.001. (**I**–**J**) Clones are marked by CD8::GFP and indicated by white dotted line. Larval brain tissues from the wild-type MARCM clones (**I**) and *snr1*^*6c*^ *hdac3*^*6c*^ MARCM clones (**J**) were implanted into the abdomen of wild-type hosts. Scale bar, 10 µm. **DOI:** http://dx.doi.org/10.7554/eLife.01906.008

To assess the involvement of histone modifications in type II neuroblast lineages, we screened a collection of 43 histone modifiers (Table 1; Kirilly et al., 2011) by RNAi under the control of a type II-specific driver but failed to identify any RNAi lines with ectopic neuroblasts. We reasoned that histone modifiers may act cooperatively with the Brm complex in type II neuroblast lineages and the phenotype may be masked due to the presence of the functional Brm complex. We therefore re-screened the same collection of potential histone modifiers in a *brm* RNAi background and showed that the simultaneous knockdown of both *brm* and *hdac3* under the control of the type II driver resulted in a more severe phenotype of ectopic neuroblasts (Figure 3D,E; 22.1 ± 7.4 neuroblasts/clone, n = 21) compared with *brm* RNAi (Figure 3B,E; 10.3 ± 5.6 neuroblasts/clone, n = 30) or *hdac3* RNAi knockdown alone (Figure 3C,E; 1.1 ± 0.5 neuroblasts/clone, n = 85). This finding suggests that HDAC3 functions cooperatively with Brm to regulate type II neuroblast lineages. Next, we took advantage of an existing deletion mutant *snr1*^*6c*^ *hdac3*^*6c*^, which removes the entire *snr1* coding region and the C-terminal region of *hdac3*. Type II neuroblast MARCM clones from *snr1*^*6c*^ *hdac3*^*6c*^ homozygotes possessed a large number of ectopic neuroblasts (Figure 3G,H, 22 ± 11.5 neuroblasts/clone, 83.0%, n = 22). The number of mature Dpn^+^ Ase^+^ INPs in each *snr1*^*6c*^ *hdac3*^*6c*^ type II neuroblast clone was modestly reduced to 15.6 ± 4.4 (n = 22) compared with 20.3 ± 3/clone (n = 20) in control, while the number of Dpn^−^ PntP1^+^ immature and early mature INPs (6.3 ± 3/clone, n = 21) are slightly greater compared with the control clones (4.1 ± 0.9, n = 21). In contrast, type I neuroblast clones of this double mutant appeared normal, as there was only one neuroblast per clone (Figure 3—figure supplement 1D, n = 17). Similar to *hdac3* RNAi (Figure 3C), neither type I nor type II neuroblast mutant clones of a loss-of-function *hdac3*^*N*^ allele had ectopic neuroblasts (Figure 3—figure supplement 1E and data not shown). Despite that ectopic neuroblasts were observed in multiple *snr1* RNAi lines (Figure 1—figure supplement 1D), *snr1*^*R3*^ MARCM clones did not show obvious ectopic type II neuroblasts (Figure 1—figure supplement 1E). Because Snr1 protein was speculated to have extended perdurance in somatic clones of its null allele (Marenda et al., 2004), the lack of phenotype in *snr1*^*R3*^ is likely due to protein perdurance in the neuroblast clones. Our data suggest that HDAC3 acts cooperatively with the Brm complex to suppress the formation of ectopic type II neuroblasts. To ascertain whether *snr1*^*6c*^ *hdac3*^*6c*^ causes tumorigenesis, larval brain tissues carrying *snr1*^*6c*^ *hdac3*^*6c*^ MARCM clones were transplanted into the abdomen of wild-type hosts. A significant portion of the mutant tissue (Figure 3J; 21%, n = 14) proliferated massively and formed malignant tumors, whilst control clones did not proliferate after the implantation (Figure 3I; n = 25). In subsequent rounds of transplantation, 70% (T1, n = 10) and 80% (T2, n = 5) of the *snr1*^*6c*^ *hdac3*^*6c*^ mutant brain tissues developed tumors, suggesting that *snr1*^*6c*^ *hdac3*^*6c*^ can induce malignant tumor-like growth after allograft culture.

**Table 1.** Histone modifiers and their RNAi lines **DOI:** http://dx.doi.org/10.7554/eLife.01906.007

S/No.	Gene name	Full name	CG #	Main function	VDRC RNAi lines
1	enok	Enoki mmushroom	CG11290	HAT	KK108400, GD37527
2	nej	Nejire/CBP	CG15319	HAT	KK105115
3	CG1894		CG1894	HAT	GD41575, GD41574
4	CG2051		CG2051	HAT	GD33458
5	Mof	Males absent on the first	CG3025	HAT	KK105370
6	Rpb4	Rpb4	CG33520	HAT	GD21985, GD23308
7	Pcaf	Gcn	CG4107	HAT	KK108943, GD21786
8	YL-1	YL-1	CG4621	HAT	GD21903
9	Chm	Chameau	CG5229	HAT	KK105542
10	Dik	Diskette	CG7098	HAT	GD46320
11	lid	Little imaginal discs	CG9088	HAT	GD42203, KK103830
12	Ada2b		CG9638	HAT	GD24076
13	Sirt7		CG11305	HDAC	GD18043, GD18045
14	HDAC4		CG1770	HDAC	GD20522
15	HDAC3		CG2128	HDAC	KK107073
16	HDACX		CG31119	HDAC	KK108098
17	Sirt4		CG3187	HDAC	GD40295, KK110639
18	Sirt2		CG5085	HDAC	KK103790
19	Sir2		CG5216	HDAC	GD23199, KK108241, KK105502
20	Bin1	Bicoid interacting protein	CG6046	HDAC	KK105352, GD15710
21	Sirt6		CG6284	HDAC	GD22483
22	Gug	Grunge	CG6964	HDAC	GD13687
23	Rpd3	HDAC1	CG7471	HDAC	GD46929, GD30600, GD46929
24	Sin3a		CG8815	HDAC	KK105852
25	Rtf1		CG10955	Methyl transferase	KK110392
26	Vig2		CG11844	Methyl transferase	KK107081, GD17245
27	egg	eggless	CG12196	Methyl transferase	KK101677, GD33730
28	esc	extra sexcombs	CG14941	Methyl transferase	GD5690, GD5692
29	set2		CG1716	Methyl transferase	GD30707
30	g9a		CG2995	Methyl transferase	GD25474
31	pr-set7		CG3307	Methyl transferase	KK105422
32	trr	trithorax-related	CG3848	Methyl transferase	GD10749, KK110276
33	CG40351		CG40351	Methyl transferase	GD40683, GD10833, GD45267
34	CG4565		CG4565	Methyl transferase	GD5665
35	mes-4		CG4976	Methyl transferase	GD10836
36	Art4	Arginine methyl transferase 4	CG5358	Methyl transferase	KK107009
37	Su(var)3–9	auppressor of variegation 3–9	CG6476	Methyl transferase	GD39377
38	Art1	Arginine methyl transferase 11	CG6554	Methyl transferase	GD40388, KK110391
39	ash2	absent, small or homeotic discs 2	CG6677	Methyl transferase	KK100718
40	LKR	Lysine ketoglutarate reductase	CG7144	Methyl transferase	GD51346
41	Su(z)12	Suppressor of Zeste 205	CG8013	Methyl transferase	GD42422, GD42423
42	Su(var)205	Suppressor of variegation 205	CG8409	Methyl transferase	KK107477
43	Ash1	Absent, small or homeotic discs 1	CG8887	Methyl transferase	GD28928

**Figure 3—figure supplement 1.**
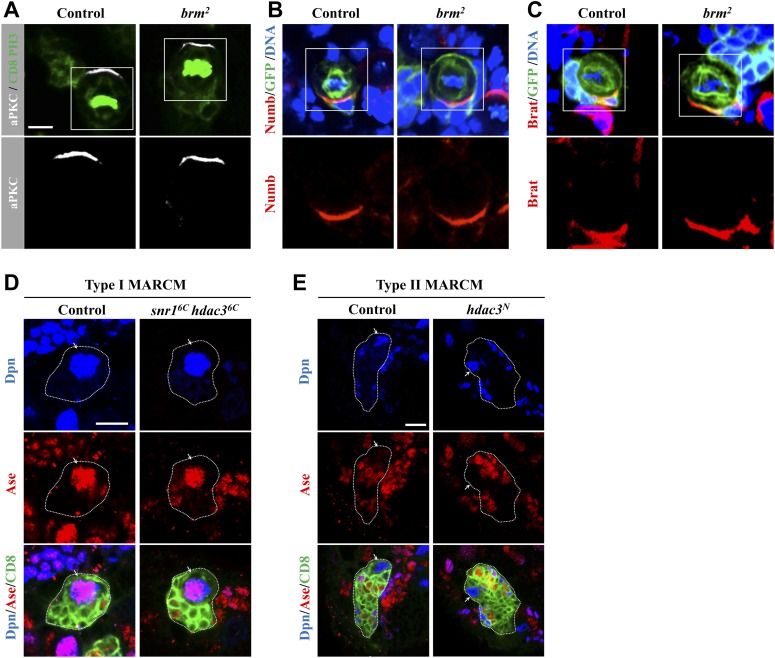
Brm is not important for the apical-basal polarity of neuroblasts. (**A**–**C**) Neuroblast of control MARCM clones and *brm*^*2*^ MARCM clones were co-labeled with aPKC (white), CD8 (green) and Phospho-Histone H3 (PH3; green) (**A**) or Numb, GFP and DNA (**B**) or Brat, GFP and DNA (**C**). Lower panels are enlarged images of the boxed region. (**D**) Type I MARCM clones from control (MARCM driver) and *snr1*^*6c*^ *hdac3*^*6c*^ were labeled with Dpn, Ase and CD8. (**E**) Type II MARCM clones from control (driver) and *hdac3*^*N*^ were labeled with Dpn, Ase and CD8. Arrows, neuroblasts. Scale bars, 5 µm (**A**–**C**), 10 μm (**D**–**E**). **DOI:** http://dx.doi.org/10.7554/eLife.01906.009

### The Brm remodeling complex physically associates with Erm and HDAC3

**Figure 4.**
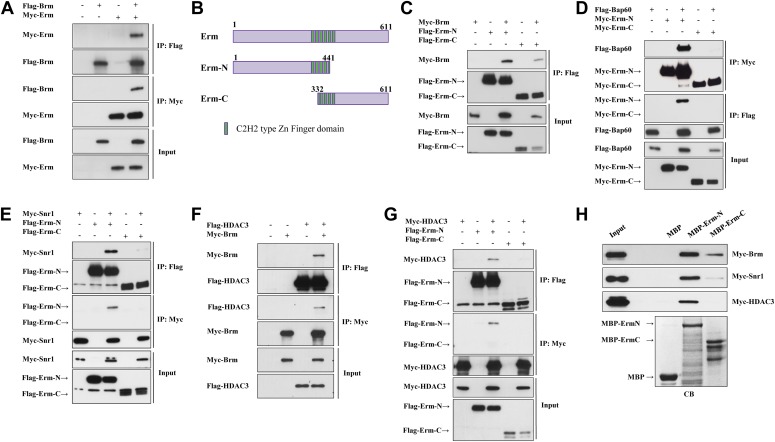
The Brm remodeling complex physically associates with Erm and HDAC3. (**A**) Co-immunoprecipitation (Co-IP) between Flag-Brm and Myc-Erm. (**B**) An illustration of Erm domains and truncated constructs. (**C**) Co-IP between Myc-Brm and Flag-Erm-N or Flag-Erm-C. (**D**) Co-IP between Flag-Bap60 and Myc-Erm-N or Myc-Erm-C. (**E**) Co-IP between Myc-Snr1 and Flag-Erm-N or Flag-Erm-C. (**F**) Co-IP was Flag-HDAC3 and Myc-Brm. (**G**) Co-IP between Flag-HDAC3 and Myc-Erm-N or Myc-Erm-C. IP was performed using anti-Flag or anti-Myc antibodies. Western blot was performed using anti-Flag and anti-Myc antibodies. (**H**) Protein pull-down assay. MBP, MBP-Erm-N and MBP-ErmC bound beads were incubated with protein extracts from S2 cells expressing Myc-Brm, Myc-Snr1 or Myc-HDAC3. Western blot was performed using an anti-Myc antibody. Coomassie blue (CB) staining showed 10% input of various purified MBP or MBP fusion proteins. **DOI:** http://dx.doi.org/10.7554/eLife.01906.010

Given that Brm is ubiquitously expressed in various cell types of larval brains, including neuroblasts, INPs, GMCs and neurons (Figure 2—figure supplement 1 and data not shown), it is conceivable that the Brm complex may associate with regulatory protein(s) or co-factor(s) that is/are specifically expressed in type II neuroblast lineages to suppress the formation of ectopic neuroblasts. We therefore assessed whether Brm could associate with two such type II-specific transcription factors, Erm and PntP1. Flag-tagged Brm was co-transfected with Myc-tagged Erm in S2 cells. Following immunoprecipitation (IP) of Flag-Brm, Erm can be specifically detected in the immune complex (Figure 4A). Consistently, Flag-Brm was detected in the immune complex following the IP of Myc-Erm (Figure 4A). In contrast, Myc-Brm did not associate with Flag-PntP1 in similar co-IP experiments (Figure 4—figure supplement 1A), suggesting that Brm specifically associates in a protein complex with Erm, but not with PntP1. Since full-length Erm had very low expression levels in S2 cells, we expressed two truncated proteins, Erm N-terminal 1-441aa (Erm-N containing N-terminal region and four of six zinc-finger domains) and Erm C-terminal 332-611aa (Erm-C containing the last four zinc-finger domains and its C-terminus), and used them for the subsequent co-IP analysis (Figure 4B). In co-IP experiments, Myc-Brm associated strongly with Flag-Erm-N and weakly with Flag-Erm-C (Figure 4C). Furthermore, we ascertained whether Brm could associate with Erm in a protein pull-down assay. Full-length Erm could not be efficiently expressed when fused with Maltose-Binding Protein (MBP) in bacteria; we therefore expressed truncated MBP-Erm-N or MBP-Erm-C. These fusion proteins were then bound to amylose resin, and subsequently incubated with protein extracts from S2 cells transfected with Myc-Brm. Following the pull-down of amylose resin, Myc-Brm associated intensely with MBP-Erm-N and weakly with Erm-C, but not with the MBP control (Figure 4H). These data suggest that Brm physically associates with Erm and Erm N-terminus appears to be more important for this association.

**Figure 4—figure supplement 1.**
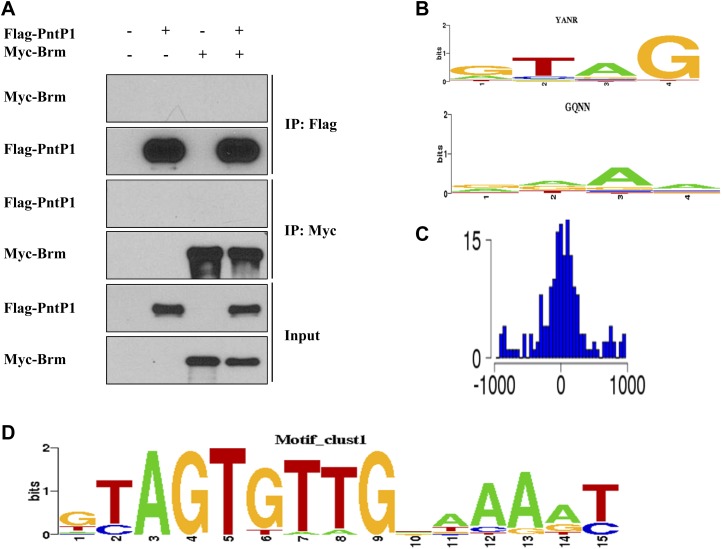
Brm and Erm may regulate gene expression of some common downstream targets. (**A**) Co-IP was performed using S2 cells expressing Flag-PntP1 and Myc-Brm. IP was performed using anti-Flag or anti-Myc antibodies. Western blot was performed using anti-Flag and anti-Myc antibodies. (**B**) The DNA binding preferences of the first zinc-finger ‘GTAG’ and the fourth zinc-finger ‘RAAA’. They are observed to be enriched in 270 Brm binding sites. (**C**) The distant distribution between the ChIP–chip peak and the occurrences of the motif in (**D**). (**D**) The de novo Erm-binding motif learned by SEME based on the 270 Brm binding sites. **DOI:** http://dx.doi.org/10.7554/eLife.01906.011

We next ascertained whether Erm associates with other components of the Brm remodeling complex, such as BAP60 and Snr1. In co-IP experiments, Flag-BAP60 was detected in the immune complex following IP of Myc-Erm-N but not Myc-Erm-C (Figure 4D). Likewise, Myc-Erm-N was detected in the immune complex when IP was performed using Flag-BAP60 (Figure 4D). Moreover, Myc-Snr1 associated with Flag-Erm-N or Flag-Erm-C in co-IP experiments (Figure 4E). Consistently, Myc-Snr1 associated with MBP-Erm-N and weakly with MBP-Erm-C in protein pull-down assays (Figure 4H). Therefore, we conclude that the several components of the Brm remodeling complex specifically associates with Erm.

Given that HDAC3 functions cooperatively with the Brm complex to suppress the generation of ectopic neuroblasts, we ascertained whether HDAC3 can physically associate with both Brm and Erm. Flag-HDAC3 and Myc-Brm were co-transfected in S2 cells. Following immunoprecipitation of Flag-HDAC3, Myc-Brm was clearly detected in the immune complex (Figure 4F). Similarly, Flag-HDAC3 was detected in the immune complex after IP of Myc-Brm (Figure 4F). Interestingly, HDAC3 also physically associates with Erm-N, but not Erm-C, in both co-IP (Figure 4G) and protein pull-down assay (Figure 4H). Taken together, these results show that Brm physically associates with Erm and HDAC3 in the protein complex.

### *brm* and *hdac3* genetically interact with *erm* to prevent dedifferentiation of INPs to neuroblasts

**Figure 5.**
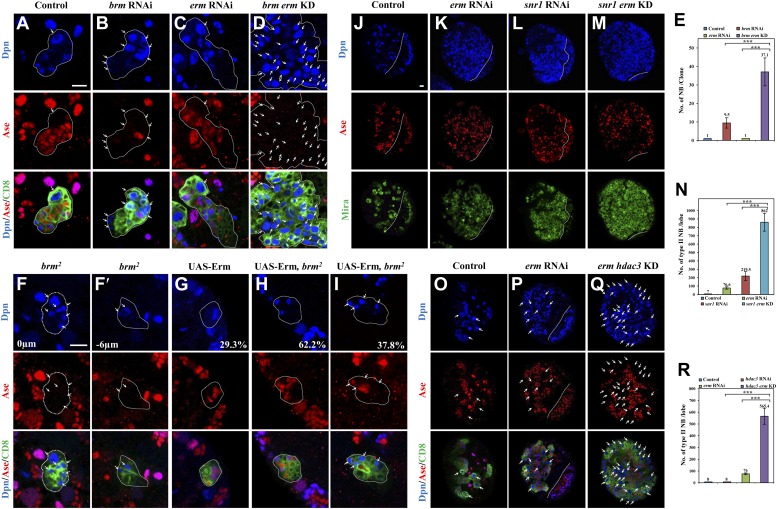
Brm genetically interacts with Erm to prevent dedifferentiation of INPs to neuroblasts. (**A**–**D**) Type II clones of control (the type II driver; **A**), *brm* knockdown (**B**), *erm* knockdown (**C**) and *brm erm* double knockdown (**D**) were labeled with Dpn (blue), Ase (red) and CD8 (green). (**E**) Quantifications of neuroblast number per type II neuroblast lineage for **A**–**D**. (**F**–**I**) Type II MARCM clones of *brm*^*2*^ (**F**, **F′**), UAS-Erm (**G**) and UAS-Erm, *brm*^*2*^ (**H**–**I**) were labeled with Dpn (blue), Ase (red) and CD8 (green). (**J**–**M**) Larval brains of control (**J**, *elav*-Gal4 driver), *erm* knockdown (**K**), *snr1* knockdown (**L**) and *erm snr1* double knockdown (**M**) were labeled with Dpn (blue), Ase (red) and Mira (green). (**N**) Quantifications of the number of type II neuroblasts per brain hemisphere in various genotypes in **J**–**M**. Control (*elav*-Gal4), 7 ± 0; *erm* RNAi, 76.6 ± 14.2; *snr1* RNAi, 219.5 ± 52.2; *erm snr1* double knockdown (KD), 862.0 ± 106.7. (**O**–**Q**) Larval brains of control (driver; **O**), *erm* knockdown (**P**) and *erm hdac3* double knockdown (**Q**) under the type II driver were labeled with Dpn (blue), Ase (red) and CD8 (green). (**R**) Quantifications of neuroblast number per brain hemisphere in **O**–**Q**. Central brain is to the left of white dotted lines. Arrows indicate neuroblasts. Clones were indicated by white dotted lines. Scale bars, 10 µm. *** indicates p<0.001. **DOI:** http://dx.doi.org/10.7554/eLife.01906.012

Given that Brm and Erm associate in a protein complex, and both of them suppress the formation of ectopic neuroblasts, we assessed whether *brm* genetically interacts with *erm* to prevent dedifferentiation of INPs to neuroblasts. First, we ascertained whether *erm* knockdown can exacerbate the *brm* RNAi phenotype in type II neuroblast lineages. The simultaneous knockdown of *brm* and *erm* resulted in a much more severe phenotype with a large number of ectopic neuroblasts in each lineage (Figure 5D,E; 37.1 ± 7.6 neuroblasts/lineage, n = 20), in contrast to *brm* knockdown alone (Figure 5B,E; 9.5 ± 2.8, n = 32). It was reported that in *erm*^*-*^ mutants, dedifferentiated neuroblasts can establish ectopic type II neuroblast lineages and form ectopic glial chambers (Weng et al., 2010). Presumably due to incomplete knockdown of *erm* that only led to weak ectopic type II neuroblasts phenotypes, *erm* RNAi under the type II driver resulted in ectopic type II lineages with each lineage containing one type II neuroblast (Figure 5C,E; 1 neuroblast/lineage, n = 60). This dramatic enhancement suggests that *brm* and *erm* genetically interact to prevent the dedifferentiation of INPs back to neuroblasts. Furthermore, knock down of *erm* by RNAi in the *brm*^*T362*^ MARCM clones (Figure 5—figure supplement 1A,B; 9.9 ± 5.5 neuroblasts/clone, n = 32) also significantly enhanced neuroblast overgrowth compared with *brm*^*T362*^ clones (Figure 5—figure supplement 1A,B; 4.1 ± 2.4 neuroblasts/clone, n = 30). However, the size of the *brm*^*T362*^ clones with *erm* knockdown remained smaller than the control clones, probably due to the reduced number of INPs that are required to expand the clonal size. Erm overexpression has previously been shown to result in premature differentiation of type II neuroblasts (Weng et al., 2010). Similarly, we found that overexpression of Erm in type II MARCM clones caused 100% of the neuroblasts to undergo premature differentiation; 29.3% of the clones contained a neuroblast that gained Ase expression and 41.5% of the clones contained a neuroblast that had gained Ase expression with strongly reduced Dpn expression, while the rest of the clones showed no obvious neuroblasts (Figure 5G and data not shown; n = 41). To assess whether this effect is dependent on its association with Brm, we overexpressed Erm in *brm*^*2*^ type II neuroblast clones. The premature differentiation of type II neuroblasts was dramatically suppressed in a *brm* loss-of-function mutant background, as there were still 37.8% of clones contained ectopic neuroblasts (Figure 5I), while 62.2% of type II neuroblasts underwent premature differentiation (Figure 5H; n = 37). Similarly, the simultaneous knockdown of both *erm* and *snr1* resulted in a more severe phenotype of ectopic neuroblasts (Figure 5M–N; 862 ± 106.8 neuroblasts/brain lobe, n = 20) compared with either *erm* knockdown (Figure 5K,N; 76.6 ± 14.2 neuroblasts/brain lobe, n = 20) or *snr1* knockdown (Figure 5L,N; 219.5 ± 52.2 neuroblasts/brain lobe, n = 20). Thus, we conclude that *brm* and *snr1* genetically interact with *erm* to prevent dedifferentiation of INPs to neuroblasts. Furthermore, the simultaneous knockdown of *hdac3* and *erm* under the control of type II driver also resulted in a more dramatic increase of ectopic type II neuroblasts (Figure 5Q,R; 565.4 ± 68.1 type II neuroblasts/brain lobe, n = 20) compared with either *erm* knockdown (Figure 5P,R; 76.0 ± 7.7 type II neuroblasts/brain lobe, n = 20) or *hdac3* knockdown (Figure 5R; 8 type II neuroblasts/brain lobe, n = 20), suggesting that *hdac3* and *erm* genetically interact in type II neuroblast lineages. Taken together, these results indicate that Brm, HDAC3, and Erm function as a repressor complex to prevent INP dedifferentiation into type II neuroblasts.

**Figure 5—figure supplement 1.**
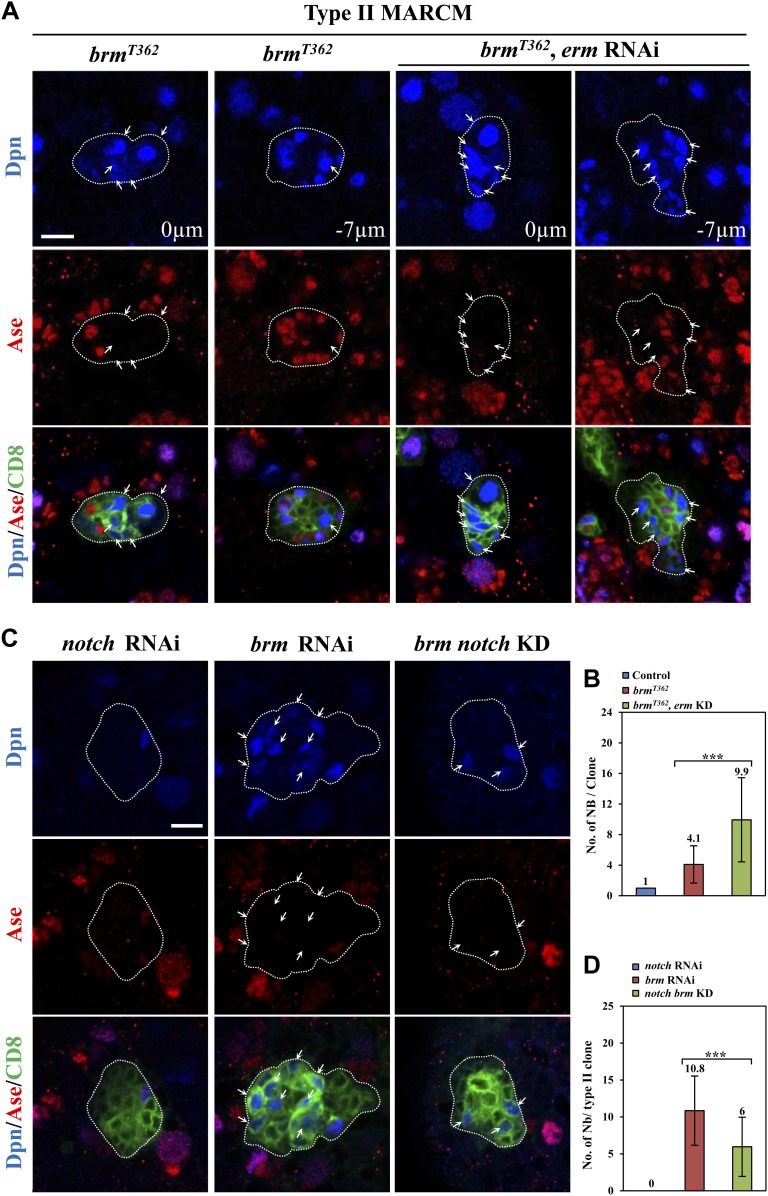
Knocking down of *erm* enhanced the neuroblast overgrowth observed in *brm* mutants. (**A**) Type II MARCM clones of *brm*^*T362*^ and UAS-Dcr2; *erm* RNAi, *brm*^*T362*^ were labeled with Dpn, Ase and CD8. Arrows, neuroblasts. Clone outline is indicated by white dotted line. Scale bar, 10 µm. (**B**) Quantification of neuroblast number per type II MARCM clone. MARCM Control, 1.0 ± 0; *brm*^*T362*^, 4.1 ± 2.4; *erm* RNAi, *brm*^*T362*^, 9.9 ± 5.5. (**C**) Simultaneous knockdown of *brm* and *notch* in type II neuroblast lineages partially suppressed the ectopic neuroblast phenotype, compared with *brm* knockdown alone. Type II neuroblast lineages are labeled with Dpn, Ase and CD8. (**D**) Quantification of genotypes in **C**. *brm notch* double knockdown, 6.0 ± 4.7 neuroblasts/lineage, n = 76; *brm* knockdown, 10.8 ± 4 neuroblasts/lineage, n = 39. *** indicates p<0.001. **DOI:** http://dx.doi.org/10.7554/eLife.01906.013

## Discussion

Here, we report a critical function of the *Drosophila* Brm remodeling complex in suppressing the formation of ectopic type II neuroblasts in larval brains. Mutants of major components of the Brm complex, including Brm and Bap55, and RNAi targeting of several Brm components formed ectopic type II neuroblasts. Therefore, the *Drosophila* Brm remodeling complex displays a tumor suppressor-like function in larval brains. Multiple subunits of the SWI/SNF complex are associated with various cancers. BAP47 (homologous to Snr1) is a bona fide tumor suppressor and the gene is deleted in pediatric rhabdoid tumors (Reisman et al., 2009). Mutations in epigenetic regulators are found in approximately half of hepatocellular carcinoma and bladder cancers, and represent a significant portion of mutated genes in medulloblastoma (Gui et al., 2011; Fujimoto et al., 2012; Pugh et al., 2012). *Drosophila* Brm complex is essential for intestinal stem cell proliferation and commitment in the adult intestine (Jin et al., 2013; Zeng et al., 2013). Two other chromatin remodeling factors, Iswi and Domino control germline stem cell and somatic stem cell self-renewal in the ovary (Xi and Xie, 2005).

We have demonstrated that Brm physically associates with Erm, a type II-specific transcription factor that prevents the dedifferentiation of INPs back into neuroblasts. Furthermore, Bap60 and Snr1, two other components of the Brm complex, also physically associate with Erm in a protein complex. Therefore, we have provided the first molecular link during the regulation of type II neuroblast lineages. We speculate that the association with Erm may provide functional specificity of the Brm remodeling complex in type II neuroblast lineages. We have also shown that *brm* genetically interacts with the type II-specific transcription factor *erm*. Ectopic neuroblast phenotype resulting from *brm* knockdown was dramatically enhanced by simultaneous knockdown of *erm*. Furthermore, *brm* knockdown, similar to *erm*^*−*^ (Weng et al., 2010), can be partially suppressed by loss of *notch* (Figure 5—figure supplement 1C,D). These functional data suggest that Erm is a co-factor of the Brm remodeling complex in type II neuroblast lineages. However, it is uncertain how the Brm–Erm protein complex functions to prevent dedifferentiation in type II neuroblast lineages.

Our bioinformatic analysis has identified a 14 bp-long motif as the de novo Erm DNA-binding motif (Figure 4—figure supplement 1B–D; and Supplementary methods) and 202 sites out of the 270 known genomic loci harboring Brm (Negre et al., 2011) also contain the de novo Erm DNA-binding motif (Table 2, Gene list). As there are many genes that are potentially co-occupied by Brm and Erm, it is possible that Brm–Erm complex results in a unique configuration of the chromatin ‘landscape’ in INPs to prevent INP dedifferentiation into neuroblasts. Therefore, disruption of chromatin remodelers may cause widespread changes to the transcriptome, thus amplifying the effect of the single genetic mutation.

**Table 2.** Predicted common target genes of Brm and Erm **DOI:** http://dx.doi.org/10.7554/eLife.01906.014

S/No.	CG name	Gene name
1	CG10033	for
2	CG10117	ttv
3	CG10137	CG10137
4	CG10159	BEAF-32
5	CG10388	Ubx
6	CG10610	ECSIT
7	CG1071	E2f2
8	CG10844	RyR
9	CG1100	Rpn5
10	CG11228	hpo
11	CG11309	CG11309
12	CG11589	VhaM9.7-c
13	CG12165	Incenp
14	CG12321	CG12321
15	CG12333	CG12333
16	CG12387	zetaTry
17	CG12797	Ciao1
18	CG12818	CG12818
19	CG12819	sle
20	CG12855	HPS1
21	CG12994	CG12994
22	CG13004	CG13004
23	CG13016	CG13016
24	CG13018	CG13018
25	CG13117	CG13117
26	CG1322	zfh1
27	CG13316	Mnt
28	CG13350	Ctf4
29	CG13366	CG13366
30	CG13432	qsm
31	CG13472	CG13472
32	CG13688	Ipk2
33	CG13900	CG13900
34	CG13919	CG13919
35	CG14291	CG14291
36	CG14463	CG14463
37	CG1453	Klp10A
38	CG14813	deltaCOP
39	CG14814	CG14814
40	CG14938	crol
41	CG14939	CycY
42	CG15010	ago
43	CG15027	CG15027
44	CG15120	CG15120
45	CG15387	CG15387
46	CG15701	CG15701
47	CG15706	CG15706
48	CG15845	Adf1
49	CG1600	Drat
50	CG1616	dpa
51	CG17033	elgi
52	CG17035	GXIVsPLA2
53	CG17052	obst-A
54	CG17233	CG17233
55	CG17249	CG17249
56	CG17259	CG17259
57	CG17260	CG17260
58	CG1765	EcR
59	CG17803	CG17803
60	CG1785	CG1785
61	CG1817	Ptp10D
62	CG18292	CG18292
63	CG1845	Br140
64	CG18660	Nckx30C
65	CG18675	CG18675
66	CG2004	CG2004
67	CG2019	disp
68	CG2051	CG2051
69	CG2146	didum
70	CG2189	Dfd
71	CG2446	Amun
72	CG2698	CG2698
73	CG2720	Hop
74	CG2813	cold
75	CG2977	Inx7
76	CG3059	NTPase
77	CG3127	Pgk
78	CG31481	pb
79	CG3157	gammaTub23C
80	CG3165	CG3165
81	CG3166	aop
82	CG31712	CG31712
83	CG31713	Apf
84	CG3178	Rrp1
85	CG31794	Pax
86	CG31852	Tap42
87	CG31855	CG31855
88	CG31911	Ent2
89	CG32022	CG32022
90	CG32556	chas
91	CG32592	hiw
92	CG33116	CG33116
93	CG33162	SrpRbeta
94	CG3587	CG3587
95	CG3666	Tsf3
96	CG3842	CG3842
97	CG3857	CG3857
98	CG3920	Reph
99	CG42254	CG42254
100	CG42311	grh
101	CG42334	comm3
102	CG42362	CG42362
103	CG42363	CG42363
104	CG42365	CG42365
105	CG42379	CG42379
106	CG42380	CG42380
107	CG42381	CG42381
108	CG4400	CG4400
109	CG4590	Inx2
110	CG4619	CG4619
111	CG4645	CG4645
112	CG4798	l(2)k01209
113	CG4996	CG4996
114	CG5229	chm
115	CG5393	apt
116	CG5505	scny
117	CG5548	CG5548
118	CG5588	Mtl
119	CG5599	CG5599
120	CG5611	CG5611
121	CG5613	CG5613
122	CG5824	l(3)07882
123	CG5836	SF1
124	CG6022	Cchl
125	CG6202	Surf4
126	CG6218	CG6218
127	CG6235	tws
128	CG6241	CG6241
129	CG6272	CG6272
130	CG6322	U4-U6-60K
131	CG6343	ND42
132	CG6401	CG6401
133	CG6511	CG6511
134	CG6556	cnk
135	CG6565	CG6565
136	CG6604	H15
137	CG6634	mid
138	CG6829	Ark
139	CG6948	Clc
140	CG6951	CG6951
141	CG6983	CG6983
142	CG7082	papi
143	CG7085	l(2)s5379
144	CG7186	SAK
145	CG7191	CG7191
146	CG7372	CG7372
147	CG7379	CG7379
148	CG7564	CG7564
149	CG7597	Cdk12
150	CG7632	CG7632
151	CG7685	CG7685
152	CG7734	shn
153	CG7771	sim
154	CG7828	APP-BP1
155	CG7845	CG7845
156	CG7849	CG7849
157	CG7957	MED17
158	CG7961	alphaCop
159	CG8067	CG8067
160	CG8241	pea
161	CG8287	Rab8
162	CG8360	CG8360
163	CG8372	CG8372
164	CG8396	Ssb-c31a
165	CG8409	Su(var)205
166	CG8481	CG8481
167	CG8790	Dic1
168	CG8798	Lon
169	CG8817	lilli
170	CG9042	Gpdh
171	CG9054	Ddx1
172	CG9063	Rich
173	CG9065	CG9065
174	CG9243	CG43345
175	CG9243	CG43346
176	CG9244	Acon
177	CG9249	CG9249
178	CG9250	Mpp6
179	CG9305	CG9305
180	CG9376	CG9376
181	CG9473	MED6
182	CG9596	CG9596
183	CG9635	RhoGEF2
184	CG9641	CG9641
185	CG9730	mRpL21
186	CG9750	rept
187	CG9829	poly
188	CG9865	CG9865

Most class I HDACs are recruited into large multi-subunit co-repressor complexes for maximal activity (Wen et al., 2000). HDAC1 and 2 are found in multiple co-repressor complexes, while to date HDAC3 appears to be uniquely recruited to the Silencing mediator of retinoic and thyroid receptors (SMRT)/Nuclear receptor co-repressor (N-CoR) complex (Guenther et al., 2000; Li et al., 2000). Here, we report that *Drosophila* HDAC3 is recruited to a novel multi-subunit complex containing Brm and Erm and that this co–repressor complex prevents dedifferentiation of INPs into type II neuroblasts. The SMRT complex appears not to be important for type II neuroblasts, as knockdown of *smrter* that encodes a core component of the SMRT complex (Heck et al., 2012) neither resulted in any ectopic type II neuroblasts nor enhanced the phenotype of ectopic neuroblasts by *brm* knockdown (data not shown). We also showed that HDAC3 dramatically enhanced the phenotype of ectopic neuroblast upon loss of *brm* or *snr1*, two core components of the Brm complex. By identifying this novel repressor complex, we have provided a mechanistic link between transcriptional repression and histone deacetylation during the suppression of dedifferentiation. HDACs are typically recruited by oncogenic protein complexes in lymphoma and leukemia and HDAC3 inhibitors are synergistic or additive with anticancer agents for therapeutics (Dokmanovic et al., 2007). Our finding that HDAC3 functions cooperatively with the Brm complex in suppressing suppressing dedifferentiation of INPs into neuroblasts and induces tumors in the allograph transplantation revealed an unexpected potential involvement of HDAC3 in tumor suppression in brain tissue. It will be of interest to determine whether this effect is conserved in the mammalian central nervous system and whether it occurs in tissues other than the brain.

## Materials and methods

### Fly stocks and antibodies

The following flies were used in this study: *brm*^*T362*^ is from J Treisman; *erm*^*1*^, *erm*^*2*^, UAS-*Erm*^*CTHA*^, UAS-*Brm* (AK Dingwall), 9D11-Gal4 (*erm*-Gal4; GM Rubin). *brm*^*2*^, *bap55*^*LL05905*^, Erm RNAi (#26778; BDSC) are from Bloomington *Drosophila* stock center. VDRC RNAi lines used: Brm (GD37720 and 37721GD), Bap60 (KK103634), Snr1 (KK108599, GD12645, and BDRC#32372), Bap55 (GD24704), Moira (GD6969), Bap180 (KK108618), dMi-2 (KK107204), nurf301 (GD46645), Acf1 (GD33446) and ISWI (GD24505). The type II neuroblast driver: w; UAS-Dicer 2, *wor*-Gal4, *ase*-Gal80/CyO; UAS-mCD8-GFP/TM3, Ser (Neumuller et al., 2011).

The primary antibodies used were: guinea-pig anti-Dpn (1:1000, J Skeath), anti-Insc (1:1000); rabbit anti-aPKCζ C20 (1:100; Santa Cruz Biotechnologies, Dallas, TX); guinea-pig anti-Numb (1:1000; J Skeath); mouse anti-Mira (1:50; F Matsuzaki); rat anti-CD8 (1:250; Caltag laboratories, United Kingdom); rabbit anti-GFP (1:500; Molecular Probes, Eugene, OR); rabbit anti-Asense (1:1000; YN Jan); rabbit anti-PntP1 (1:100; J Skeath); rabbit anti-Brm (1:100; L Zhang); rat anti-phospho-Histone H3 (1:1000; Cell Signaling, Danvers, MA); rabbit anti-phospho-Histone H3 (1:200; Sigma, St Louis, MO); mouse anti-dMyc (1:5; B Edgar). Antibodies for western blotting used were: mouse anti-Myc (1:2000; Abcam, United Kingdom) and mouse anti-Flag (1:1000; Sigma).

### Immunohistochemistry and immunoblotting

Third instar larval brains were dissected and fixed with 3.7% formaldehyde in PBS. Fixed brains were blocked with 3% BSA for one hour and then incubated with primary antibody in 3% BSA (in 0.3% PBS-T) over night at 4°C. Following three times washing (10 min each), larval brains were incubated with secondary antibody diluted in 0.3% PBS-T for 1.5 hr. After two times washing (10 min each), DNA was labeled by ToPro-3 (1:5000; Invitrogen, Carlsbad, CA) in 0.3% PBS-T for 20 min. Larval brains were mounted in vector shield (Vector Laboratory, Burlingame, CA) for confocal microscopy. Images were obtained using a Zeiss LSM 700 confocal microscope and processed with Adobe Photoshop CS5.1.

### Clonal analysis

MARCM clones were generated as previously described (Lee and Luo, 1999). Briefly, larvae were heat shocked at 37°C for 90 min at 24 hr ALH and at 10–16 hr after the first heat shock. Larvae were further aged for 3 days at 25°C, and larval brains were dissected and processed for immunohistochemistry. To generate type II neuroblast clones, UAS lines were crossed to the type II driver at 25°C and shifted to 29°C at 24 hr ALH. Wandering third instar larvae were dissected after incubation for 3 or 4 days at 29°C.

### S2 cell culture, transfection and co-immunoprecipitation

*Drosophila* S2 cells were cultured in Shields and Sang M3 insect medium (Sigma-Aldich), and supplemented with 10% fetal bovine serum (FBS; Hyclone, Logan, UT). Flag-Erm or Myc-Brm generated by Gateway cloning was transfected into S2 cells using Effectene Transfection Reagent (QIAGEN, The Netherlands). S2 cells were collected 48 hr after transfection for protein homogenization. 80 μg S2 cells are homogenized with lysis buffer (25 mM Tris pH8/27.5 mM NaCl/20 mM KCl/25 mM sucrose/10 mM EDTA/10 Mm EGTA/1 mM DTT/ 10% (vol/vol) glycerol/0.5% Nonidet P40) with Proteases inhibitors (Complete, Boeringher; PMSF 10 μg/ml, Sodium orthovanadate 10 μg/ml). The supernatants were used for immunoprecipitation with anti-Myc or anti-Flag for overnight at 4°C, followed by incubation with protein A/G beads for two hours (Pierces, Rockford, IL). Protein A/G beads were washed with cold PBS for three times. Bound proteins were separated by SDS-PAGE and analyzed by western blotting.

### Protein pull-down assay

MBP or MBP fusion proteins were expressed in BL21 cells and bound on amylose resin (Cart# E8021L; NEW ENGLAND Biolabs Inc., United Kingdom). 50 μg of purified MBP fusion proteins bound on amylose resin were incubated for 3 hr at 4°C with protein extracts from 100 μg S2 cells that were homogenized in lysis buffer with proteases inhibitors. After washing amylose resin three times for 7 min each with 1 ml lysis buffer, bound proteins were separated by SDS-PAGE and analyzed by western blotting.

### Transplantation

Allograft culture of larval brain tissue was carried out as previously described (Castellanos et al., 2008). Third instar larval brains are dissected and the tissue is cut into pieces. A piece of tissue is collected with the tip of a glass needle and injected in the mid-ventral abdomen of a young female fly.

### Generation of plasmid constructs

Plasmid constructs were generated using either pENTR Directional TOPO Cloning Kit (Invitrogen) or In-Fusion HD Cloning Kit (Clontech, Mountain View, CA). ESTs used in this study were GH14092 (Erm), LD36356 (Brm), LD09078 (Bap60), GH08712 (Snr1) (*Drosophila* Genomics Resource Centre [DGRC], Bloomington, IN). Briefly, coding region of genes were amplified by PCR, inserted into the pENTR/D-TOPO vector (Invitrogen) and destination vectors (pAMW or PAFW) were generated by LR recombination.

### Bioinformatics

From the previously reported ChIP–chip data, we obtained a list of 270 Brm binding sites. To determine if Erm also binds to these binding sites, we first analyzed the DNA binding domains of Erm, which contain 6 zinc fingers. Each zinc-finger domain was assigned a DNA binding preference (position weighted matrix) based on published methods (Kaplan et al., 2005). We then scanned these six DNA binding preferences of approximately +/−200 bp around the 270 Brm binding sites and found the DNA binding preferences of the 1st zinc-finger ‘GTAG’ and the 4th zinc-finger ‘RAAA’ are enriched in the 270 Brm binding sites. The sites enriched with these two binding preferences were subjected to further analysis using the de novo motif-finding program SEME (Zhang et al., 2013), and a 14 bp-long motif was identified as the de novo Erm DNA-binding motif. We scanned +/−200 bp around the 270 Brm binding sites with the de novo Erm DNA-binding motif. Among them, 202 sites (FDR<0.0001) were identified as putative Erm-binding sites with an AUC score of 0.73, which is significantly higher than the AUC score computed for random motifs (0.5). As a negative control, the same approach was also applied to predict the motif of Zinc-finger protein (Zif), which regulates asymmetric division of neuroblasts and therefore is unlikely to be a co-factor with Brm. The predicted Zif DNA-binding motif differs dramatically with the predicted Erm DNA-binding motif in sequence. Furthermore, it was not significantly enriched in the 270 Brm binding sites and had an AUC score of 0.54, similar to the AUC score (0.5) for random motifs. Thus, our data suggests that Brm and Erm can potentially regulate a set of common downstream targets.
